# Using contrast-enhanced CT and non-contrast-enhanced CT to predict *EGFR* mutation status in NSCLC patients—a radiomics nomogram analysis

**DOI:** 10.1007/s00330-021-08366-y

**Published:** 2021-11-22

**Authors:** Xiaoyan Yang, Min Liu, Yanhong Ren, Huang Chen, Pengxin Yu, Siyi Wang, Rongguo Zhang, Huaping Dai, Chen Wang

**Affiliations:** 1 Department of Pulmonary and Critical Care Medicine, China-Japan Friendship Hospital, Capital Medical University, Beijing, China; 2 National Center for Respiratory Medicine, Institute of Respiratory Medicine, Chinese Academy of Medical Sciences, National Clinical Research Center for Respiratory Diseases, Beijing, China; 3 Department of Radiology, China-Japan Friendship Hospital, Beijing, China; 4 Department of Clinical Pathology, China-Japan Friendship Hospital, Beijing, China; 5 Infervision Medical Technology Co Ltd, Beijing, China; 6 Chinese Academy of Medical Sciences and Peking Union Medical College, Beijing, China

**Keywords:** Non-small cell lung cancer, Epidermal growth factor receptor, Radiomics, Tomography, X-ray computed

## Abstract

**Objectives:**

To develop and validate a general radiomics nomogram capable of identifying *EGFR* mutation status in non-small cell lung cancer (NSCLC) patients, regardless of patient with either contrast-enhanced CT (CE-CT) or non-contrast-enhanced CT (NE-CT).

**Methods:**

A total of 412 NSCLC patients were retrospectively enrolled in this study. Patients’ radiomics features not significantly different between NE-CT and CE-CT were defined as general features, and were further used to construct the general radiomics signature. Fivefold cross-validation was used to select the best machine learning algorithm. Finally, a general radiomics nomogram was developed using general radiomics signature, and clinical and radiological characteristics. Two groups of data collected at different time periods were used as two test sets to access the discrimination and clinical usefulness. Area under the receiver operating characteristic curve (ROC-AUC) was applied to performance evaluation.

**Result:**

The general radiomics signature yielded the highest AUC of 0.756 and 0.739 in the two test sets, respectively. When applying to same type of CT, the performance of general radiomics signature was always similar to or higher than that of models built using only NE-CT or CE-CT features. The general radiomics nomogram combining general radiomics signature, smoking history, emphysema, and ILD achieved higher performance whether applying to NE-CT or CE-CT (test set 1, AUC = 0.833 and 0.842; test set 2, AUC = 0.839 and 0.850).

**Conclusions:**

Our work demonstrated that using general features to construct radiomics signature and nomogram could help identify *EGFR* mutation status of NSCLC patients and expand its scope of clinical application.

**Key Points:**

*• General features were proposed to construct general radiomics signature using different types of CT of different patients at the same time to identify EGFR mutation status of NSCLC patients.*

*• The general radiomics nomogram based on general radiomics signature, and clinical and radiological characteristics could identify EGFR mutation status of patients with NSCLC and outperformed the general radiomics signature.*

*• The general radiomics nomogram had a wider scope of clinical application; no matter which of NE-CT and CE-CT the patient has, its EGFR mutation status could be predicted.*

**Supplementary Information:**

The online version contains supplementary material available at 10.1007/s00330-021-08366-y.

## Introduction

Considering the growing insight into the molecular mechanisms of lung cancer, the treatment of non-small-cell lung cancer (NSCLC) has shifted its focus to determining oncogenic driver mutation subtypes. The most common gene mutation in NSCLC is epidermal growth factor receptor (*EGFR*) mutation [1, 2]. A recent study showed that the patient who received third-generation EGFR-TKI of osimertinib even had a longer overall survival [3]. Therefore, the testing of *EGFR* mutation status before treatment is very important.

The detection of *EGFR* mutant status relies on tumor tissue from surgical or tissue biopsy, which is an invasive sampling method. Inspired by genomics and tumor heterogeneity, radiomics approach transforms any type of medical images into quantitative data to guide clinical decisions [4, 5]. Studies have shown that either non-contrast-enhanced CT (NE-CT) or contrast-enhanced CT (CE-CT) can be used to build radiomics models to predict *EGFR* mutation status [6–9]. CE-CT showed superior diagnosis abilities over NE-CT in identifying the type of *EGFR* mutant [10]. Radiomics comparison study showed that some radiomics features were not significantly different between NE-CT and CE-CT [11]. However, whether radiomic features extracted from NE-CT and CE-CT can be used together to build *EGFR* mutation status prediction model remains unknown.

The purpose of this study is to develop a method to construct radiomics signature and nomogram using different types of CT from different patients simultaneously, so that it cannot only identify *EGFR* mutation status of NSCLC patients, but also can be applied to different types of CT.

## Materials and methods

### Study population

This study was approved by our institutional review board. Informed consent from the patients was waived for this retrospective study. The study comprises two sets of patients from different study time. The first set included a total of 784 patients with pathological confirmed NSCLC from January of 2017 to June 2019. The second set is from June of 2020 to December of 2020, when a total of 44 patients with NSCLC and who underwent paired NE-CT and CE-CT before surgery were retrospectively enrolled. The exclusion criteria were as follows: (I) without molecular testing of *EGFR* mutation; (II) without CT before surgery; (III) with a history of other malignant tumors; (IV) with therapy before detection of *EGFR* mutations. The *EGFR* mutant status was detected with a polymerase chain reaction and confirmed by direct sequencing [12]. The clinical information including age, gender, smoking history, pulmonary function tests, pathological type, and the results of molecular testing were collected from the personal medical charts.

**Fig. 1:**
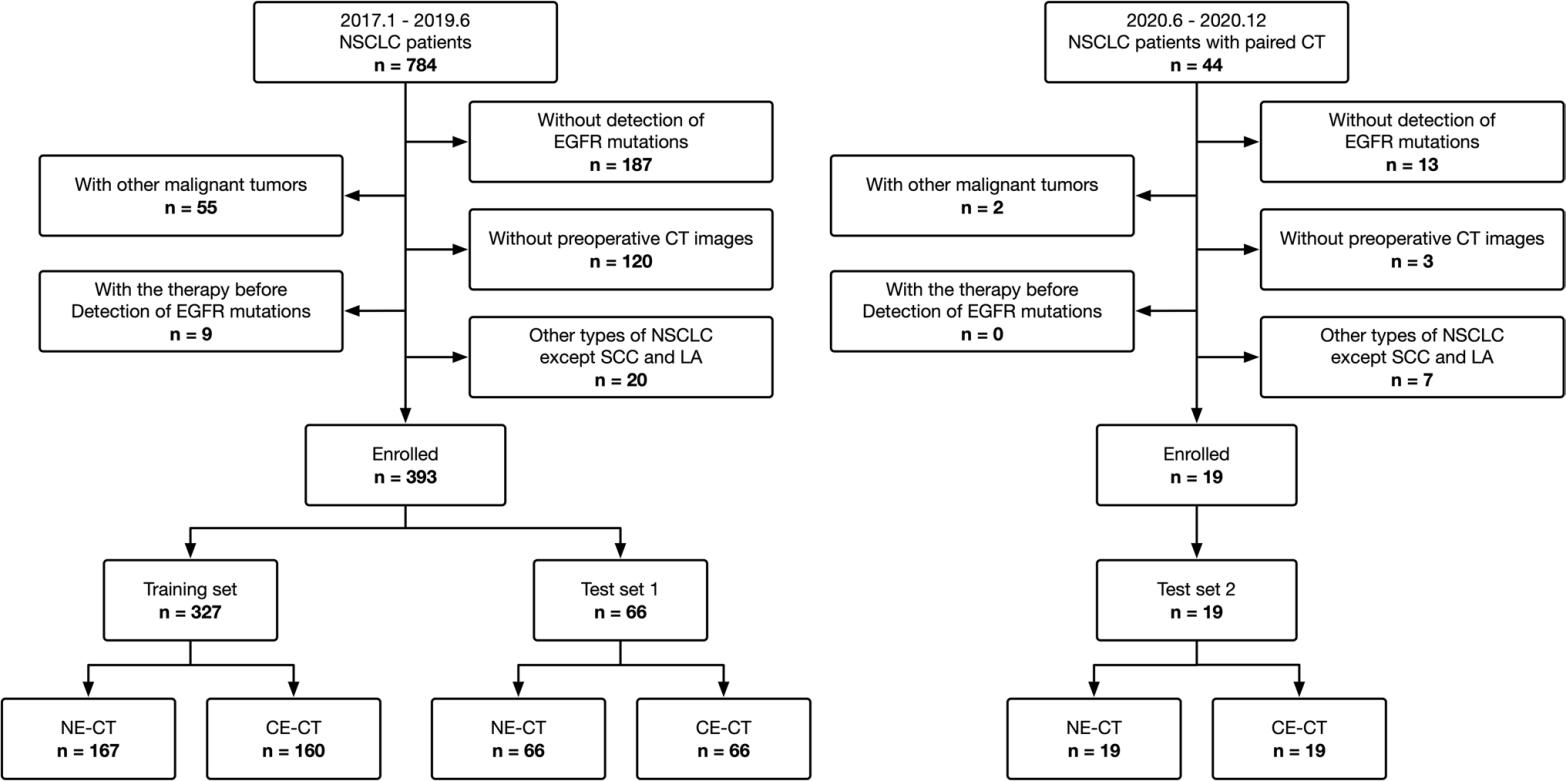
The flowchart of inclusion and exclusion criteria of patients to this study

As shown in Fig. 1, the enrolled patients were divided into three sets: (I) training set (*n* = 327), which consisted of patients whose scan time was before June of 2019 and only had one type of CT (167 NE-CT and 160 CE-CT); (II) test set 1 (*n* = 66), which consisted of patients whose scan time was before June of 2019 and had both NE-CT and CE-CT (66 NE-CT and 66 CE-CT); (III) test set 2 (*n* = 19), which consisted of patients whose scan time was during June of 2020 and December of 2020 and had both NE-CT and CE-CT (19 NE-CT and 19 CE-CT).

### CT scan protocol

All patients who underwent chest CT scan used one of the two multidetector CT systems (Brilliance CT, Philips Healthcare; Toshiba CT). All the subjects were examined at full inspiration in the supine position with or without contrast material. The scanning parameters were as follows: 100–120 kVp, 100 mA, detector collimation of 64 × or 128 × 0.625 mm; field of view of 350 × 350 mm; and matrix of 512 × 512 using a reconstruction kernel for the lung. For CE-CT, after routine CT, a dose of 85 mL non-ionic iodinated contrast material (350 mg iodine/mL, Omnipaque, GE Healthcare) was injected into the antecubital vein at a rate of 3.0 mL/s using an automated injector (Ulrich CT Plus 150, Ulrich Medical). CT scanning was performed again with a 25-s delay after the injection. NE-CT and CE-CT of 5 mm were retrieved from the Picture Archiving and Communication System (PACS) workstation with format of DICOM.

### CT evaluation

One chest radiologist with 15 years of experience interpreted the CT radiological features, including size, location, mass or nodules, morphology, opacity of tumor, interstitial changes of lung, bronchitis, bronchiectasis, emphysema, lymphadenopathy, pleural thickening, and pleural retraction. She described the location of tumor in the five lobes consisting of the upper lobes, middle lobe, and lower lobes. The radiological morphology features of tumor were subcategorized as lobulation, spiculation, cavitation, and pleural retraction. The opacity of tumor was classified into solid, part-solid, mostly part-solid, or ground-glass nodule [13]. Interstitial lung disease (ILD) diagnosis referred to the diagnostic criteria updated by the American Thoracic Society (ATS)/European Respiratory Society (ERS) in 2013 [14].

### VOI segmentation and radiomics features extraction

**Fig. 2:**
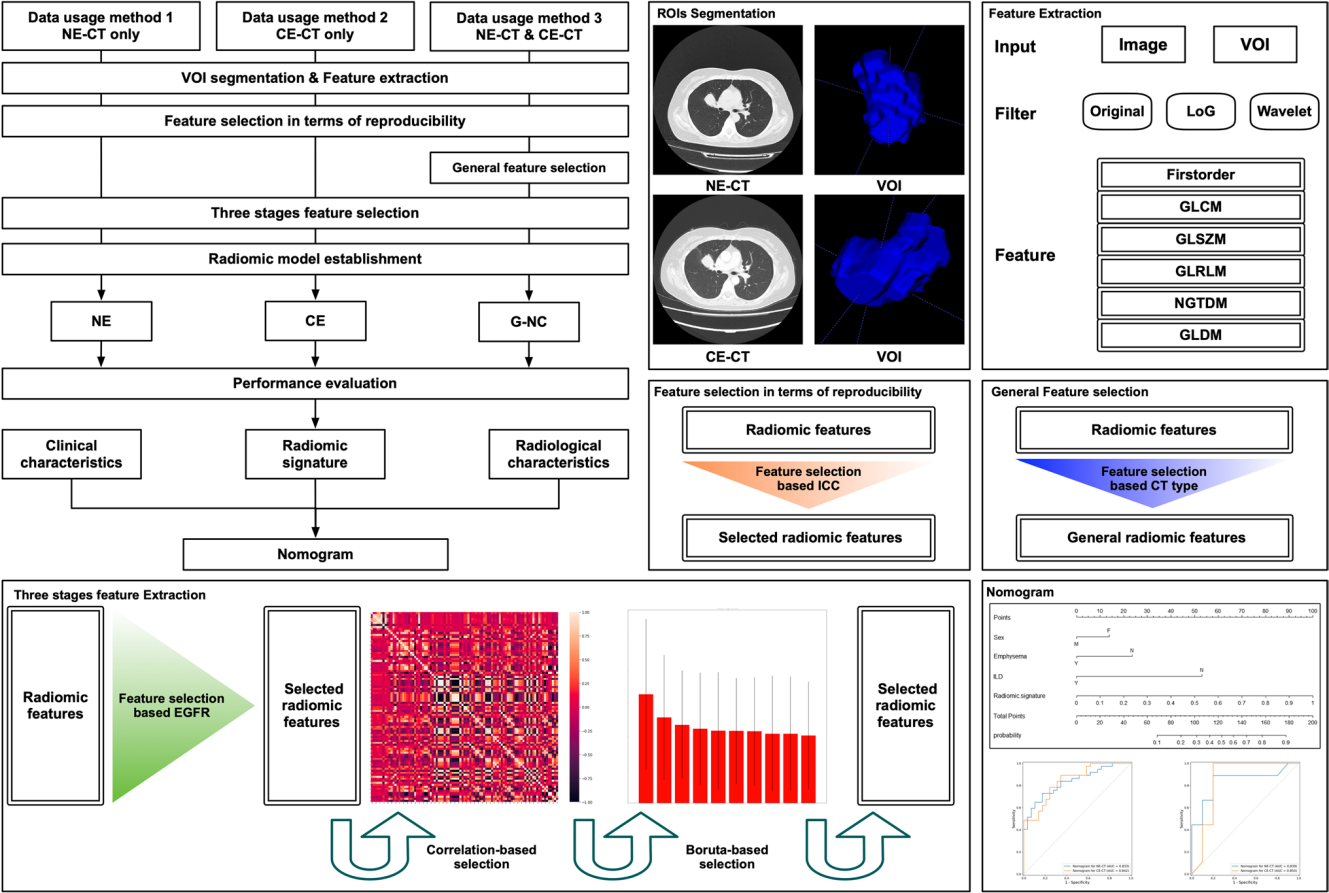
The workflow of this study

Figure 2 showed the workflow of this study. The entire tumor on CT images was defined as the volume of interest (VOI). The same chest radiologist used a research platform (InferScholar; https://www.infer-vision.com/) to manually segment the VOI slice. the voxel size of VOI was resampled to 1*1*1 mm^3^ by cubic interpolation to reduce the variability of radiomic feature values due to different voxel sizes [15].

For each VOI, PyRadiomics [16] was used to automatically extract the quantitative radiomic features. Radiomic features extraction was performed on the VOI and the VOI converted by different filters. Specifically, six types of radiomics features were extracted, including first-order (18 features), gray-level co-occurrence matrix (GLCM, 22 features), gray-level size zone matrix (GLSZM, 16 features), gray-level run length matrix (GLRLM, 16 features), neighboring gray-tone difference matrix (NGTDM, 5 features), and gray-level dependence matrix (GLDM, 14 features). When calculating the texture features and two first-order features, the values of CT were discretized with a fixed bin width of 32 Hounsfield units (HU). Overall, 1092 quantitative radiomic features were extracted. Details of the radiomics features were provided in supplementary Table 1.

### Feature selection in terms of reproducibility

To evaluate the reproductivity of the radiomic features, 1 month later, 50 patients were randomly selected from the training set and re-segmented by the previous radiologist. Intraclass correlation coefficient (ICC) was utilized to evaluate the intra-observer agreement between the two repeated segmented; values > 0.80 indicated good reproducibility, otherwise was excluded in the following feature selection process[8].

### General feature

To explore whether the radiomic features obtained by NE-CT and CE-CT can be used simultaneously for analysis, we compared three data usage methods. Here, we proposed a general features approach, called G-NC (General NE-CT and CE-CT). The general features selection that contained three steps was applied in the training set. The first step was to divide the data into two groups according to their CT type (NE-CT or CE-CT). In the second step, the differences of radiomic features extracted from two groups were compared using Mann–Whitney *U* test. In the third step, the radiomic features showed significant differences between two groups (*p* < 0.05) were excluded, and the rest of the features were retained as general features. The second data usage method was called NE, which indicated the radiomic features extracted from NE-CT. The last data usage method was to use the radiomic features extracted from CE-CT, namely CE.

### Selection of prediction factors and establishment of prediction model

We used a three-stage feature selection method for predicting factors selection. The first stage was to perform a Mann–Whitney *U* test between *EGFR* wild-type and the *EGFR* mutant patients for all radiomic features. The features with *p* < 0.05 were input to the second stage. The second stage was to calculate the Pearson correlation coefficient, defined as *r*, between each pair of input features. If there was a pair of features with |*r*|> 0.85, only the one with a smaller *p* value calculated in the first stage would be kept. The last stage was to evaluate all remaining features’ importance by a feature selection algorithm called Boruta. The algorithm was designed as a wrapper around Random Forest classification algorithm, which provided unbiased and stable selection of important and non-important attributes from an information system. After feature selection, a fivefold cross-validation strategy was used to choose the best machine learning method to construct a radiomic model [17]. The machine learning methods used included support vector machine (SVM), logistic regression (LR), random forest (RF), gradient boosting decision tree (GBDT), and naive Bayesian classification (NBC).

### Nomogram construction

The radiomics signature was the prediction score of the radiomic model (details in supplementary). All clinical and radiological characteristics that differ significantly between *EGFR* wild-type and *EGFR* mutant in the training set, as well as the radiomics signature, were included in a multivariate logistic regression using forward stepwise selection to select independent predictors. Finally, a nomogram was contracted based on the independent predictors.

### Statistical analysis

The differences of all features between *EGFR* wild-type and *EGFR* mutant were assessed using Mann–Whitney *U* test for continuous variables and Fisher’s exact test or chi-square test for categorical variables. The discrimination was evaluated by area under the receiver operating characteristic curve (ROC-AUC). Decision curve analysis (DCA) was used to evaluate clinical usefulness by quantifying the net benefits of the nomogram in both two test sets [18]. DeLong test [19, 20] was used for statistical comparisons of ROC curves. All statistical analyses were performed with R (version 3.5.0; http://www.Rpro-ject.org) and SPSS (version 22.0, IBM). A two-tailed *p* value was considered statistically significant if less than 0.05.

## Results

### Clinical and radiological characteristics

There were 176, 37, and 9 patients with *EGFR* mutation and 151, 29, and 10 with *EGFR* wild-type in the training set, test set 1, and test set 2, respectively. *EGFR* mutation rates were significantly higher in women than those in men, and in non-smokers than in smokers. Regarding other non-radiomics features, histology, COPD, ILD (*p* < 0.001), type of lesion (*p* = 0.008), size (*p* = 0.001), and emphysema (*p* < 0.001) were statistically different between the two groups. *EGFR* mutation was more likely to be found in groups with smaller size, adenocarcinoma and without emphysema, COPD, and ILD (Table.1).

**Table 1:** Characteristics of patients in the training, test set 1, and test set 2

		Training set			Test set 1			Test set 2		
		*EGFR* +	*EGFR*-	*p* value	*EGFR* +	*EGFR*-	*p* value	*EGFR* +	*EGFR*-	*p* value
Patients, no (%)		176	151		37	29		9	10	
Clinical										
Age, mean (SD)		60.00 (10.02)	59.76 (10.81)	0.836	58.08 (8.45)	59.66 (13.17)	0.558	68.11 (7.17)	66.50 (10.01)	0.695
Sex, no. (%)	Female	120 (68.2)	61 (40.4)	< 0.001	22 (59.5)	12 (41.4)	0.145	5 (55.6)	3 (30.0)	0.370
	Male	56 (31.8)	90 (59.6)		15 (40.5)	17 (58.6)		4 (44.4)	7 (70.0)	
Smoking history, no. (%)	0	146 (83.0)	97 (64.2)	< 0.001	34 (91.9)	20 (69.0)	0.017	7 (77.8)	3 (30.0)	0.070
	1	30 (17.0)	54 (35.8)		3 (8.1)	9 (31.0)		2 (22.2)	7 (70.0)	
Histology, no. (%)	SCC	1 (0.6)	19 (12.6)	< 0.001	0 (0.0)	4 (13.8)	0.02	0 (0.0)	0 (0.0)	-
	Adenocarcinoma	175 (99.4)	132 (87.4)		37 (100.0)	25 (86.2)		9 (100.0)	10 (100.0)	-
COPD, no. (%)	0	145 (82.4)	104 (68.9)	0.004	26 (70.3)	19 (65.5)	0.681	6 (66.7)	8 (80.0)	0.628
	1	31 (17.6)	47 (31.1)		11 (29.7)	10 (34.5)		3 (33.3)	2 (20.0)	
Radiological										
Size (mm), mean (SD)		19.40 (11.37)	25.11 (19.12)	0.001	23.92 (14.62)	31.10 (20.67)	0.103	24.56 (13.33)	31.4 (18.09)	0.366
Location, no. (%)	Left upper lobe	44 (25.0)	46 (30.5)	0.278	10 (27.0)	7 (24.1)	0.879	1 (11.1)	2 (20.0)	0.795
	Left lower lobe	28 (15.9)	27 (17.9)		5 (13.5)	5 (17.2)		3 (33.3)	2 (20.0)	
	Right upper lobe	62 (35.2)	41 (27.2)		14 (37.8)	10 (34.5)		4 (44.4)	3 (30.0)	
	Right middle lobe	17 (9.7)	9 (5.9)		3 (8.2)	1 (3.5)		0 (0.0)	0 (0.0)	
	Right lower lobe	25 (14.2)	28 (18.5)		5 (13.5)	6 (20.7)		1 (11.1)	3 (30.0)	
Type of lesion, no. (%)	nodule	151 (85.8)	112 (74.2)	0.008	25 (67.6)	15 (51.7)	0.191	8 (88.9)	7 (70.0)	0.582
	mass	25 (14.2)	39 (25.8)		12 (32.4)	14 (48.3)		1 (11.1)	3 (30.0)	
Opacity, no. (%)	GGO	43 (24.4)	35 (23.2)	0.216	10 (27.0)	5 (17.2)	0.034	1 (11.1)	0 (0.0)	1.000
	Partly solid	40 (22.7)	26 (17.2)		4 (10.8)	3 (10.4)		1 (11.1)	1 (10.0)	
	Most partly solid	22 (12.5)	13 (8.6)		7 (18.9)	0 (0.0)		2 (22.2)	3 (30.0)	
	Solid	71 (40.4)	77 (51.0)		16 (43.2)	21 (72.4)		5 (55.6)	6 (60.0)	
Vacuole sign, no. (%)	0	142 (80.7)	126 (83.4)	0.517	28 (75.7)	25 (86.2)	0.286	5 (55.6)	4 (40.0)	0.656
	1	34 (19.3)	25 (16.6)		9 (24.3)	4 (13.8)		4 (44.4)	6 (60.0)	
Lobulation, no. (%)	0	88 (50)	71 (47.0)	0.591	15 (40.5)	11 (37.9)	0.830	4 (44.4)	1 (10.0)	0.141
	1	88 (50)	80 (53.0)		22 (59.5)	18 (62.1)		5 (55.6)	9 (90.0)	
Spiculation, no. (%)	0	92 (52.3)	69 (45.7)	0.236	14 (37.8)	8 (27.6)	0.381	2 (22.2)	2 (20.0)	1.000
	1	84 (47.7)	82 (54.3)		23 (62.2)	21 (72.4)		7 (77.8)	8 (80.0)	
Pleural retraction, no. (%)	0	97 (55.1)	86 (57.0)	0.738	18 (48.6)	17 (58.6)	0.420	2 (22.2)	0 (0.0)	0.211
	1	79 (44.9)	65 (43.0)		19 (51.4)	12 (41.4)		7 (77.8)	10 (10.0)	
Obstructive pneumonia, no. (%)	0	174 (98.9)	147 (93.4)	0.420	37 (100.0)	27 (93.1)	0.189	9 (100.0)	10 (10.0)	-
	1	2 (1.1)	4 (2.6)		0 (0)	2 (6.9)		0 (0.0)	0 (0.0)	-
Bronchitis, no. (%)	0	152 (86.4)	119 (78.8)	0.071	27 (73.0)	19 (65.5)	0.513	4 (44.4)	8 (80.0)	0.170
	1	24 (13.6)	32 (21.2)		10 (27.0)	10 (34.5)		5 (55.6)	2 (20.0)	
Emphysema, no. (%)	0	156 (88.6)	99 (65.6)	< 0.001	29 (78.4)	16 (55.2)	0.045	7 (77.8)	7 (70.0)	1.000
	1	20 (11.4)	52 (34.4)		8 (21.6)	13 (44.8)		2 (22.2)	3 (30.0)	
Lymphadenopathy, no. (%)	0	144 (81.8)	110 (72.8)	0.052	28 (75.7)	19 (65.5)	0.366	5 (55.6)	5 (50.0)	1.000
	1	32 (18.2)	41 (27.2)		9 (24.3)	10 (34.5)		4 (44.4)	5 (50.0)	
Pleural thickening, no. (%)	0	159 (90.3)	140 (92.7)	0.444	34 (91.9)	24 (82.8)	0.285	6 (66.7)	9 (90.0)	0.303
	1	17 (9.7)	11 (7.3)		3 (8.1)	5 (17.2)		3 (33.3)	1 (10.0)	
Bronchiectasis, no. (%)	0	161 (91.5)	132 (87.4)	0.230	35 (94.6)	25 (86.2)	0.392	4 (44.4)	7 (70.0)	0.370
	1	15 (8.5)	19 (12.6)		2 (5.4)	4 (13.8)		5 (55.6)	3 (30.0)	
ILD, no. (%)	0	174 (98.9)	131 (86.7)	< 0.001	37 (100.0)	23 (79.3)	0.005	9 (100.0)	8 (80.0)	0.474
	1	2 (1.1)	20 (13.2)		0 (0.0)	6 (20.7)		0 (0.0)	2 (20.0)	

*COPD*, chronic obstructive lung disease; *EGFR*, epidermal growth factor receptor; *ILD*, interstitial lung disease; *SCC*, squamous cell carcinoma

### Radiomics model analysis

The final feature counts of NE, CE, and G-NC were 5, 1, and 5, respectively (supplementary Table 2). The machine learning algorithms used for NE, CE, and G-NC were LR, LR, and RF (supplementary Table 3). The prediction performance of radiomics models was described in Table 2. Regardless of which test set, using CE-CT for prediction yielded higher performance. Compared with NE and CE, G-NC had the best prediction performance in both two test sets. In particular, when NE-CT was used for prediction in the test set 1, the performance improvement was significant (AUC: 0.656 vs. 0.730; *p* < 0.05).

**Table 2:** The predictive performance of each radiomic model on the two test sets. For the radiomic model constructed using only NE-CT or CE-CT, the performance was only evaluated on the corresponding type of CT

Model	CT type	Data number	Test set 1		Test set 2	
			NE-CT	CE-CT	NE-CT	CE-CT
NE	NE-CT	167	0.656 ± 0.018	\	0.657 ± 0.031	\
CE	CE-CT	160	\	0.737 ± 0.028	\	0.711 ± 0.035
G-NC	NE and CE-CT	327	0.730 ± 0.028	0.756 ± 0.043	0.727 ± 0.033	0.739 ± 0.023
*p* value (G-NC vs. other)			0.048	0.059	0.205	0.582

*p*: Delong test values, when using NE-CT as input, calculate the significant difference between G-NC model and NE model; when using CE-CT as input, calculate the significant difference between G-NC model and CE model

*CE-CT*, contrast-enhanced CT; *G-NC*, general NE-CT and CE-CT; *NE-CT*, non-contrast-enhanced CT

### Diagnostic performance measurements

**Fig. 3:**
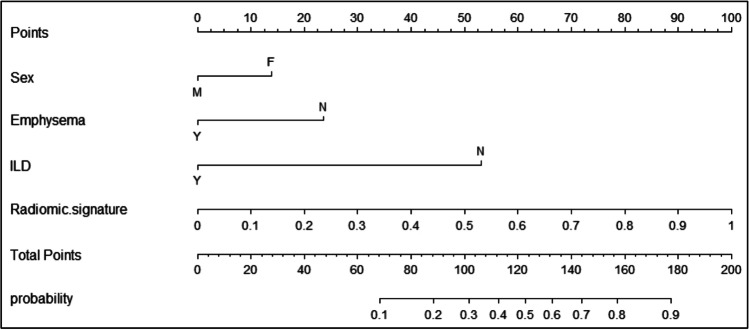
Nomogram. The nomogram was built in the training set with the sex, emphysema, ILD, and radiomic signature

As shown in Table 3 and Fig. 3, a multivariate logistic regression was used to develop an individualized prediction nomogram. Table 4 indicated the prediction performance of nomogram when different types of CT were used for prediction in the two test sets. In the test set 1, the AUCs of the nomogram were 0.833 and 0.842 (accuracy: 0.727 and 0.758; sensitivity: 0.784 and 0.784; specificity: 0.655 and 0.724) for NE-CT and CE-CT, respectively. In the test set 2, the AUCs of the nomogram were 0.839 and 0.850 on NE-CT and CE-CT, respectively. The accuracy, sensitivity, and specificity have the same values for NE-CT and CE-CT (accuracy: 0.842; sensitivity: 0.889; specificity: 0.800).

**Table 3:** Multivariable logistic regression for nomogram construction

Independent predictors	*β*	OR (95% CI)	*p*
Sex	− 0.561	0.571 (0.337–0.967)	0.037
Emphysema	− 0.950	0.387 (0.202–0.741)	0.004
ILD	− 2.141	0.118 (0.025–0.544)	0.006
Radiomic signature	4.031	56.328 (14.078–225.379)	< 0.001
Intercept	− 1.299	0.273 (0.125–0.597)	0.001

*CI*, confidence interval; *ILD*, interstitial lung disease; *OR*, odds ratio

**Table 4:** Performance of EGFR mutation predicted by nomogram in the two test sets

Performance	Test set 1		Test set 2	
	NE-CT	CE-CT	NE-CT	CE-CT
AUC	0.833 (0.737–0.918)	0.842 (0.733–0.926)	0.839 (0.641–1.000)	0.850 (0.608–1.000)
Accuracy	0.727 (0.621–0.833)	0.758 (0.652–0.848)	0.842 (0.684–1.000)	0.842 (0.684–1.000)
Sensitivity	0.784 (0.636–0.906)	0.784 (0.636–0.905)	0.889 (0.625–1.000)	0.889 (0.667–1.000)
Specificity	0.655 (0.476–0.821)	0.724 (0.548–0.885)	0.800 (0.500–1.000)	0.800 (0.500–1.000)

*AUC*, area under curve; *CE-CT*, contrast-enhanced CT; *NE-CT*, non-contrast-enhanced CT

**Fig. 4:**
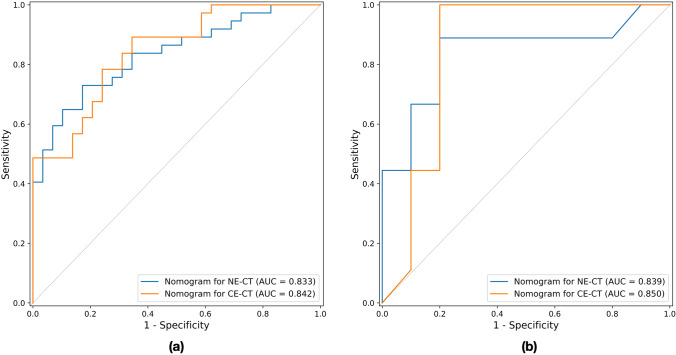
Receiver operating characteristic curves (ROCs). **a** ROC for the nomogram using NE-CT and CE-CT to predict in test set 1. **b** ROC for the nomogram using NE-CT and CE-CT to predict in test set 2

ROC curves of nomogram on the two test sets are shown in Fig. 4. On test set 1, when using NE-CT to predict, the nomogram is significantly outperformed than NE model (*p* = 0.013), and it is also significantly outperformed than G-NC model (*p* = 0.027). When using CE-CT to predict, there is no significant difference between the nomogram and the CE model, nor the G-NC model. On test set 2, no matter whether NE-CT or CE-CT is used for prediction, there is no significant difference between nomogram and other models. The correlation was used to evaluate the consistency of the nomogram in predicting NE-CT and CE-CT of the same subject. The correlation coefficients were 0.772 and 0.660 in the test set 1 and test set 2, respectively.

### Clinical use

**Fig. 5:**
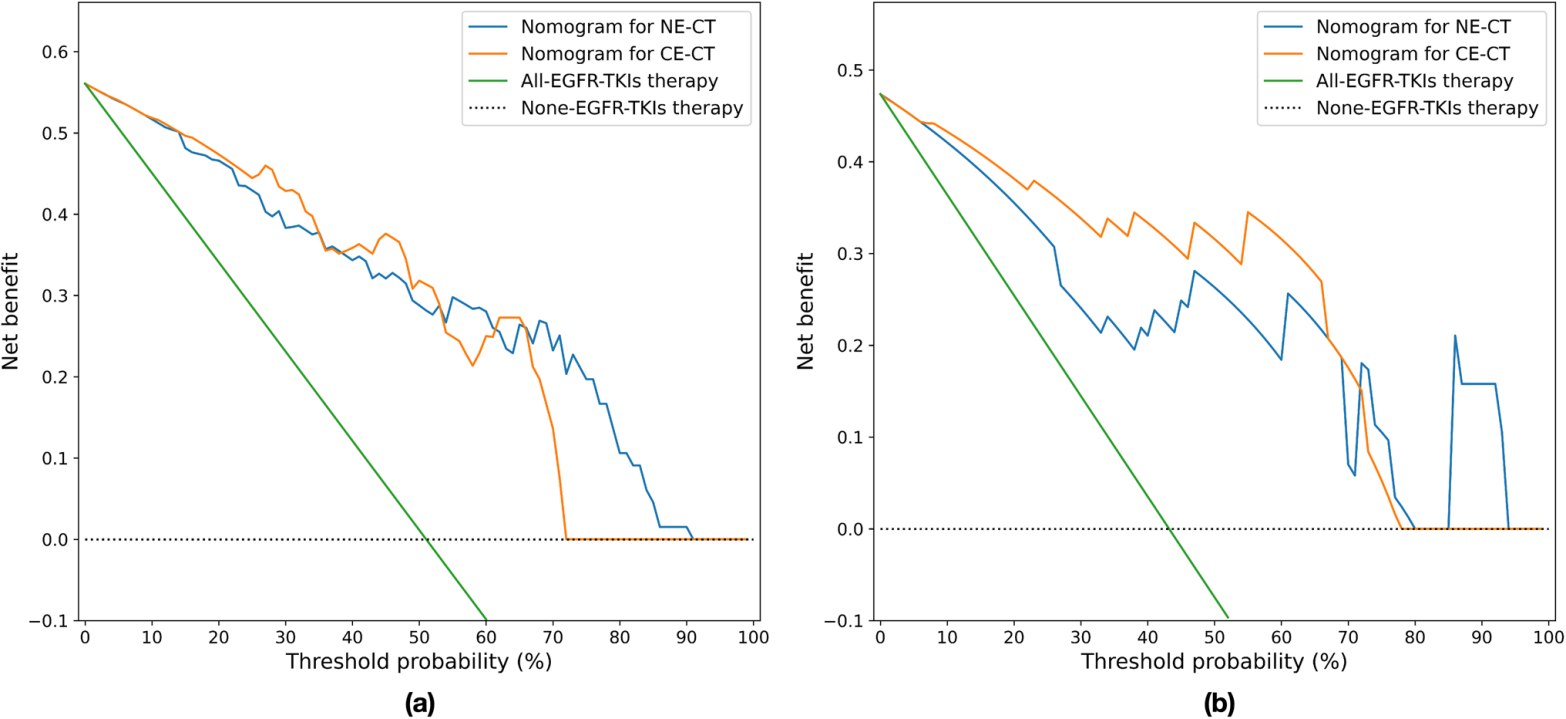
Decision curve analysis (DCA). The y axis represents the net benefit, which was determined by calculating the difference between the expected benefit and the expected harm associated with each proposed model [net benefit = true-positive rate (TPR) – (false-positive rate (FPR) × weighting factor), where the weighting factor = threshold probability/ (1-threshold probability)]. The green line represents the assumption that all patients with EGFR-TKIs therapy. The dotted line represents the assumption that all patients without EGFR-TKIs therapy. **a** DCA for the nomogram using NE-CT and CE-CT in test set 1. For threshold probabilities > 70%, nomogram using NE-CT to predict added more benefit than using CE-CT. **b** DCA for the nomogram using NE-CT and CE-CT in test set 2. For threshold probabilities < 70%, nomogram using CE-CT to predict added more benefit than using NE-CT. On both two test sets, using nomogram for prediction added more or equal benefit than using the “all-EGFR-TKIs therapy” and “none-EGFR-TKIs therapy” at any given threshold probability

The decision curve shown in Fig. 5 was used to evaluate the benefits of the nomogram using NE-CT (blue line) to predict and using CE-CT to predict (orange line). On the test set 1, for threshold probabilities > 70%, nomogram using NE-CT to predict added most benefit. On test set 2, for threshold probabilities < 70%, nomogram using CE-CT to predict added most benefit. On both two test sets, at any given threshold probability, using nomogram for prediction added more or equal benefit than using the “All-EGFR-TKIs therapy” and “None-EGFR-TKIs therapy.”

## Discussion

Radiomics can transform any type of medical images into quantitative data to aid in diagnosis and treatment, such as identifying *EGFR* mutant status. Most radiomics signatures are based on a single type of medical image, which limits the scope of their use due to uncertain image type accessibility for patients in clinical practice. Moreover, using only one type of medical image for radiomics signature development cannot tap into the advantages of other existing types of data. In this study, we proposed a method to construct radiomics signature using multiple types of CT all at once. Both our proposed general radiomics signature and nomogram could be directly applied on patient’s NE-CT or CE-CT, which further expand its scope of clinical application. The detection of *EGFR* mutation status could potentially guide physicians to treat patients with distinct therapeutic strategies. Currently, radiomics features extracted from NE-CT or CE-CT are widely used in the literature. Previous studies revealed that radiomics signature based on NE-CT had good performance in *EGFR* mutation detection with AUC of 0.796 in the test set 1 and on CE-CT showed an accuracy of 0.755 and a sensitivity of 0.929 [9]. Another study showed the performance of the radiomics signature based on CE-CT was better than that based on NE-CT, but not statistically significant [10]. Inspired by Kakino R et al. [11], we hypothesized that both NE-CT and CE-CT-based radiomics signatures can detect *EGFR* mutation. The experimental results on two test sets showed that the general radiomics signature can be applied to NE-CT and CE-CT, and the performance was better than the radiomics signature based on a single type of CT (NE and CE). Especially for NE-CT, a significant performance improvement has been obtained.

The data size of the training set for build general radiomics signature was twice the data size of training sets for building NE and CE, which may lead to incomparable performance. However, we argue that this is one of the benefits that our proposed general feature could bring, that is, we could utilize more data in a real-world clinical setting by merging different CT types, thereby improving the performance of the model. We conducted an ablation experiment to prove that the performance improvement was not only due to the increase of data size, but also because of the rational use of data (supplementary). On the one hand, directly mixing NE-CT and CE-CT for modeling did not lead to performance improvement. On the other hand, even under the same amount of data, general radiomics signature had the best performance.

Radiomics is not the only non-invasive detection method for *EGFR* mutations. Previously, physicians and radiologists tried to predict *EGFR* mutations through clinical and conventional CT characteristics, which were easily collected and would not be influenced by different CT types. Our findings are consistent with those of most existing studies that *EGFR* mutations were associated with female, non-smokers, and adenocarcinoma histology [21, 22]. Furthermore, the rate of *EGFR* mutations in NSCLC with COPD was significantly lower than that in NSCLC patients without COPD, in line with previous findings [23]. Past studies found that the radiological characteristics of maximum diameter, location, density, lymphadenopathy, spiculation, vacuole sign, air bronchograms, pleura retraction, emphysema, and fibrosis were associated with *EGFR* mutation status [24, 25]. Consistent with these studies, we found smaller size, GGO, bronchiectasis, lymphadenopathy, obstructive pneumonia and absence of emphysema, and fibrosis were more likely to be associated with tumors with *EGFR* mutations, despite insignificant difference in density, bronchiectasis, lymphadenopathy, and obstructive pneumonia. We also found that the frequency of *EGFR* mutations was higher in patients without emphysema or fibrosis than patients with emphysema or fibrosis, which is consistent with results of a prior study [26]. This indicates that CT findings of emphysema or fibrosis may be possible to predict the presence of *EGFR* mutations. Thus, a natural step forward is to combine radiomics signature and clinical or radiological characteristics to improve the accuracy of *EGFR* mutation status.

For this purpose, we constructed the general radiomics nomogram, which consists of general radiomics signature, smoking history, emphysema, and ILD. The result on the both two test sets indicated that regardless of whether NE-CT or CE-CT was used for prediction, the predictive performance of nomogram outperformed that of general radiomics signature and other models. The result of correlation matrix indicated that the nomogram predictions had good correlation for using CE-CT and NE-CT of the same subject on the test set 1 and test set 2. The decision curve analysis also showed that the nomogram added more or equal benefits than using the “All-EGFR-TKIs therapy” and “None-EGFR-TKIs therapy” at any given threshold probability.

There are several limitations in this study. First, this is a retrospective and single-center study. Although we constructed a time-based testing set in this study, external validation with more samples from other institutions is needed. Second, we divided all patients with both NE-CT and CE-CT into the test set, which may introduce sample selection bias. Third, our general feature selection method may filter out some radiomics features that may benefit the prediction of *EGFR* mutation status. In this study, we found that the feature groups used to establish NE and CE both contained wavelet-LHH_ngtdm_Strength. However, this feature was significantly different between NE-CT and CE-CT, and therefore excluded in general radiomics signature building process. It is necessary to develop a comprehensive method to use the radiomics features extracted from NE-CT and CE-CT more effectively in the future.

In conclusion, the general radiomics signature built jointly based on NE-CT and CE-CT has a good performance for *EGFR* mutation status prediction. Additionally, the nomogram consisting of radiomics, clinical, and radiological characteristics showed the most optimal predictive ability. Both general radiomics signature and nomogram are applicable to both NE-CT and CE-CT. Therefore, in clinical practice, the nomogram could be a potential non-invasive tool for *EGFR* mutation status detection.

## Supplementary Information

Below is the link to the electronic supplementary material.

Supplementary file1 (DOCX 27 KB)

## Abbreviations

ATS: American Thoracic Society
CI: 95% Confidence interval
CT: Computed tomography
CE-CT: Contrast-enhanced CT
COPD: Chronic obstructive pulmonary disease
DICOM: Digital Imaging and Communications in Medicine
EGFR: Epidermal growth factor receptor
EGFR-TKI: EGFR tyrosine kinase inhibitors
ERS: European Respiratory Society
GBDT: Gradient boosting decision tree
G-NC: General NE-CT and CE-CT
ICC: Intraclass correlation coefficient
ILD: Interstitial lung disease
LR: Logistic regression
NBC: Naive Bayesian classification
NE-CT: Non-contrast-enhanced CT
NSCLC: Non-small-cell lung cancer
PACS: Picture Achiving and Communication System
RF: Random forest
SVM: Support Vector machine
VOI: Volume of interest

## Declarations

### Guarantor

The scientific guarantor of this publication is Prof. Huaping Dai, MD.

### Statistics and biometry

No complex statistical methods were necessary for this paper.

### Methodology

• Retrospective

• Diagnostic or prognostic study

• Performed at one institution
