# Supplementary material for: Using contrast-enhanced CT and non-contrast-enhanced CT to predict EGFR mutation status in NSCLC patients—a radiomics nomogram analysis
Source: Eur Radiol. 2021 Nov 22;32(4):2693–703. doi: 10.1007/s00330-021-08366-y (PMC8921110; doi:10.1007/s00330-021-08366-y)
Supplement: Supplementary file 1 — Supplementary file1 (DOCX 27 KB) [file 330_2021_8366_MOESM1_ESM.docx]

Table.1 Different filter and radiomic features used in feature extraction.

| **Filter** | **Firstorder** | **GLCM** | **GLSZM** |
| --- | --- | --- | --- |
| Original  log-sigma-1-0-mm  log-sigma-1-5-mm  log-sigma-2-0-mm  wavelet-LLH  wavelet-LHL  wavelet-LHH  wavelet-HLL  wavelet-HLH  wavelet-HHL  wavelet-HHH  wavelet-LLL | '10Percentile',  '90Percentile',  'Energy',  'Entropy',  'InterquartileRange',  'Kurtosis',  'Maximum',  'MeanAbsoluteDeviation',  'Mean',  'Median',  'Minimum',  'Range',  'RobustMeanAbsoluteDeviation',  'RootMeanSquared',  'Skewness',  'TotalEnergy',  'Uniformity',  'Variance' | 'Autocorrelation',  'JointAverage',  'ClusterProminence',  'ClusterShade',  'ClusterTendency',  'Contrast',  'Correlation',  'DifferenceAverage',  'DifferenceEntropy',  'DifferenceVariance',  'JointEnergy',  'JointEntropy',  'Imc1',  'Imc2',  'Idm',  'Idmn',  'Id',  'Idn',  'InverseVariance',  'MaximumProbability',  'SumEntropy',  'SumSquares' | GrayLevelNonUniformity',  'GrayLevelNonUniformityNormalized',  'GrayLevelVariance',  'HighGrayLevelZoneEmphasis',  'LargeAreaEmphasis',  'LargeAreaHighGrayLevelEmphasis',  'LargeAreaLowGrayLevelEmphasis',  'LowGrayLevelZoneEmphasis',  'SizeZoneNonUniformity',  'SizeZoneNonUniformityNormalized',  'SmallAreaEmphasis',  'SmallAreaHighGrayLevelEmphasis',  'SmallAreaLowGrayLevelEmphasis',  'ZoneEntropy',  'ZonePercentage',  'ZoneVariance' |
| **GLSZM** | **NGTDM** | **GLDM** |  |
| GrayLevelNonUniformity',  'GrayLevelNonUniformityNormalized',  'GrayLevelVariance',  'HighGrayLevelZoneEmphasis',  'LargeAreaEmphasis',  'LargeAreaHighGrayLevelEmphasis',  'LargeAreaLowGrayLevelEmphasis',  'LowGrayLevelZoneEmphasis',  'SizeZoneNonUniformity',  'SizeZoneNonUniformityNormalized',  'SmallAreaEmphasis',  'SmallAreaHighGrayLevelEmphasis',  'SmallAreaLowGrayLevelEmphasis',  'ZoneEntropy',  'ZonePercentage',  'ZoneVariance' | 'Busyness',  'Coarseness',  'Complexity',  'Contrast',  'Strength' | 'DependenceEntropy',  'DependenceNonUniformity',  'DependenceNonUniformityNormalized',  'DependenceVariance',  'GrayLevelNonUniformity',  'GrayLevelVariance',  'HighGrayLevelEmphasis',  'LargeDependenceEmphasis',  'LargeDependenceHighGrayLevelEmphasis',  'LargeDependenceLowGrayLevelEmphasis',  'LowGrayLevelEmphasis',  'SmallDependenceEmphasis',  'SmallDependenceHighGrayLevelEmphasis',  'SmallDependenceLowGrayLevelEmphasis' |  |

Table.2 Final feature used for different model construction.

| **Model** | **Features** |
| --- | --- |
| NE | wavelet-LHH_ngtdm_Strength  wavelet-LHH_gldm_DependenceEntropy  wavelet-LLL_glszm_LargeAreaLowGrayLevelEmphasis  wavelet-LLL_firstorder_Minimum  wavelet-LLH_ngtdm_Contrast |
| CE | wavelet-LHH_ngtdm_Strength |
| M-NC | log-sigma-1-5-mm-3D_firstorder_Kurtosis  wavelet-LHL_glcm_ClusterShade  wavelet-LHH_ngtdm_Strength  wavelet-LLL_glszm_LargeAreaLowGrayLevelEmphasis  wavelet-LLH_firstorder_Mean  original_ngtdm_Contrast |
| G-NC | original_firstorder_Kurtosis  log-sigma-1-5-mm-3D_firstorder_Kurtosis  wavelet-LLL_ngtdm_Contrast  original_glcm_MaximumProbability  wavelet-LLL_glszm_LargeAreaLowGrayLevelEmphasis |

Table.3 The five-fold cross-validation predictive performance of all machine learning methods based on different data usage in the training set.

| Model | CT type | Data number | SVM | LR | RF | GBDT | NBC |
| --- | --- | --- | --- | --- | --- | --- | --- |
| NE | NE-CT | 167 | 0.44±0.08 | 0.69±0.08 | 0.65±0.06 | 0.60±0.07 | 0.67±0.08 |
| CE | CE-CT | 160 | 0.57±0.11 | 0.74±0.12 | 0.68±0.08 | 0.73±0.10 | 0.71±0.10 |
| M-NC | NE & CE-CT | 327 | 0.64±0.09 | 0.64±0.03 | 0.65±0.04 | 0.61±0.03 | 0.63±0.03 |
| G-NC | NE & CE-CT | 327 | 0.69±0.04 | 0.68±0.03 | 0.73±0.06 | 0.69±0.06 | 0.72±0.06 |

**Radiomics signature**

Because we use five-fold cross-validation for training on the training set, we can get five models. For each sample in the training set, we use the prediction score obtained when it is used as the cross-validation verification group as its radiomics signature. For each sample in the test set 1 and test set 2, we use the average of the prediction scores of the five models as its radiomics signature.

**Ablation experiment**

We conducted an ablation experiment to prove that the performance improvement is not only due to the increase in the size of data, but because of the rational use of data. Firstly, we performed 100 random sampling experiments, and each time we will randomly sample 160 data from the training set to build G-NC. Except for the difference in the size of data, the other processes are the same as in the manuscript. The cross-validation results on the training set are the average of 100 experimental results. Secondly, we directly mixing NE-CT and CE-CT for modeling, called M-NC (Mixed NE-CT and CE-CT). The five-fold cross-validation predictive performance was shown in supplementary Table.4.

| Model | CT type | Data number | SVM | LR | RF | GBDT | NBC |
| --- | --- | --- | --- | --- | --- | --- | --- |
| G-NC* | NE & CE-CT | 160 | 0.60±0.05 | 0.63±0.05 | 0.70±0.04 | 0.66±0.04 | 0.69±0.04 |
| M-NC | NE & CE-CT | 327 | 0.64±0.09 | 0.64±0.03 | 0.65±0.04 | 0.61±0.03 | 0.63±0.03 |

Table.4 The five-fold cross-validation predictive performance of all machine learning methods based on different data usage in the training set.

* indicated the random sampling experimental.

For the 100 random sampling experiments, the evaluation results on the two test sets are the average of 100 experimental results. For the M-NC, the evaluation results on the two test sets are the average of five trained model results. The result was shown in supplementary Table.5.

Table.5 The predictive performance of radiomic models on the two test sets.

|  | | | Test set 1 | | Test set 2 | |
| --- | --- | --- | --- | --- | --- | --- |
| Model | CT type | Data number | NE-CT | CE-CT | NE-CT | CE-CT |
| NE | NE-CT | 167 | 0.656±0.018 | \ | 0.657±0.031 | \ |
| CE | CE-CT | 160 | \ | 0.737±0.028 | \ | 0.711±0.035 |
| G-NC* | NE & CE-CT | 160 | 0.704±0.022 | 0.737±0.022 | 0.689±0.025 | 0.728±0.022 |
| M-NC | NE & CE-CT | 327 | 0.668±0.017 | 0.712±0.019 | 0.678±0.032 | 0.717±0.023 |

* indicated the random sampling experimental.

On the one hand, directly mixing NE-CT and CE-CT for modeling does not bring about performance improvement. On the other hand, even under the same amount of data, general radiomics signature has best performance.
